# Current challenges and future directions for brain age prediction in children and adolescents

**DOI:** 10.1038/s41467-025-63222-7

**Published:** 2025-08-20

**Authors:** Lucy Whitmore, Dani Beck

**Affiliations:** 1 Department of Psychology, University of Oregon, Eugene, OR USA; 2 Department of Psychology, PROMENTA Research Center, University of Oslo, Oslo, Norway; 3 Division of Mental Health and Substance Abuse, Diakonhjemmet Hospital, Oslo, Norway

**Keywords:** Neuroscience, Development of the nervous system

## Abstract

Advancements in computational techniques have enhanced our understanding of human brain development, particularly through high-dimensional data from magnetic resonance imaging (MRI). One notable approach is the *brain-age prediction* framework, which predicts biological age from neuroimaging data and calculates the brain age gap (BAG), a marker of deviation from chronological age. Most commonly applied to adult samples, this approach is now increasingly used in children and adolescents. However, several considerations must be taken into account when applying brain-age prediction in youth. In this Perspective, we outline important challenges and provide recommendations for researchers as well as future directions for the field.

## Abstract (web summary)

This Perspective outlines how brain-age prediction models are increasingly applied to child and adolescent populations and examines the specific challenges this entails. It offers practical recommendations and future directions for future research in the field.

## Introduction

The human brain undergoes profound structural and functional changes throughout childhood and adolescence^1^. Neuroimaging advances over the last 25 years have established reproducible patterns of brain development^2^. Morphometric studies report reduced gray matter volume, monotonic cortical thinning, and surface area increases in childhood, followed by decreases in adolescence^3^. White-matter volume progressively increases throughout childhood and adolescence, mirrored by higher fractional anisotropy (FA) and lower mean diffusivity (MD), indicating microstructural changes measured by diffusion tensor imaging (DTI)^4^. Functional connectivity strength changes from childhood to adulthood, with an increase in network integration (stronger within-network connectivity) and segregation (weaker between-network connectivity)^5^. Overall, brain development in childhood and adolescence involves complex, dynamic changes reflecting reorganization and optimization^6^ that is shaped by the interplay of genetics and the environmental context.

**Fig. 1:**
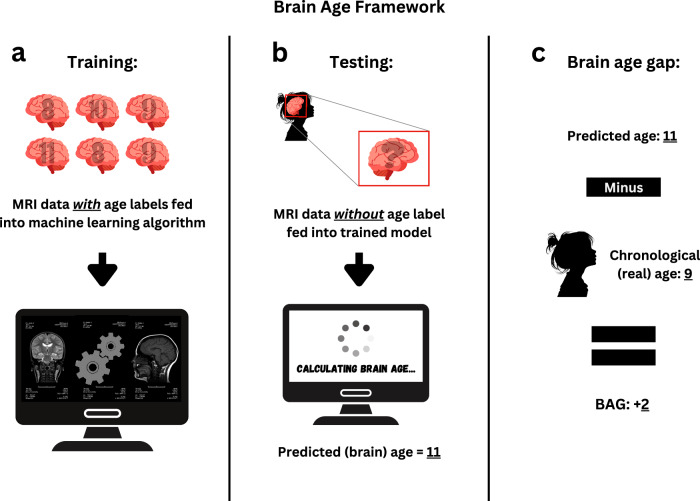
Brain-age prediction. The illustration shows the processes of **a** training, **b** testing, and **c** calculation of brain age gap (BAG) for one individual in the brain age framework.

While MRI offers high spatial resolution, providing detailed information on the structure and function of the human brain, this information is multidimensional and complex, resulting in different brain characteristics being examined in isolation. Machine learning reduces this complexity by building statistical models of the brain based on MRI datasets. One example is brain-age prediction, which reduces brain MRI features into a summary score reflecting normative brain health and integrity^7^. To calculate brain age, researchers train models on large MRI datasets from individuals across different ages. The model learns patterns that predict age from brain characteristics and is then applied to new scans. By comparing predicted and chronological ages, researchers can assess deviations from typical age-related brain structure (see Fig. 1).

The difference between brain-predicted age and chronological age, termed brain age gap (BAG), indexes this deviation. A higher brain age than chronological age indicates an older-looking brain, often interpreted as accelerated maturation in childhood and adolescence^8^, and as decline in adulthood and senescence^9^. Inversely, a lower brain age may indicate delayed maturation in youth and potentially better brain health in adulthood and senescence^10^.

Numerous studies have linked inter-individual variation in BAG to multiple phenotypes, including cognitive functioning^11^, cardiometabolic health^12–15^, lifestyle factors^16^, mental disorders^17,18^, and neurodegenerative disorders (e.g., stroke, Alzheimer’s)^19,20^. However, most studies have focused on adult populations, rely on cross-sectional designs, and primarily use T1-weighted MRI data. Recent years have seen a slow but steady increase in brain-age prediction application in youth samples, but this expansion brings forth a new set of challenges that necessitate thorough discussion with its propagators and the neuroimaging field at large. The dynamic nature of age-related brain changes during youth poses significant challenges in interpreting BAG. Brain-age models may collapse complex, overlapping developmental patterns into one metric^21^. In a unidimensional model, delayed or accelerated development in different regions can average out. Further concerns include differences in imaging modalities, scanner acquisitions, and model features^19,22^, applying models outside their training ranges, limited longitudinal data, and ignoring key maturational processes (e.g., pubertal development^16^) or socio-environmental factors (e.g., early-life adversity^23^).

The objective of this Perspective is to discuss the advancement of brain-age prediction for child and adolescent research. We define childhood according to the pediatric staging in the AAP Bright Futures guidelines^24^ and the Centers for Disease Control and Prevention developmental milestone framework^25^, treating childhood as roughly 3–9 years (spanning early and middle-childhood stages). We define adolescence according to the World Health Organization^26^ as 10–19 years. We use youth as an umbrella term covering both periods. Reviewing all studies is not within the scope of this paper, but we will outline key similarities, limitations, and offer recommendations for the field. We also discuss key methodological challenges when applying brain-age models to child and adolescent cohorts, while acknowledging that some of these challenges overlap with adult and lifespan issues.

## Overview of existing literature

Brain-age prediction has been investigated in relation to a number of domains in youth, including mental health, genetics, physical development, cognition, and environmental factors. Here, we briefly review key findings in the literature. For methodological details (e.g., samples, models, features), see Supplementary Table 1.

### Mental health

A number of different mental health outcomes have been related to both positive and negative BAGs in youth. Negative BAGs have been related to generalized anxiety^27^, Autistic Spectrum Disorder symptom severity^28^, attention deficit hyperactivity disorder symptoms^29^, elevated Child Behavior Checklist scores^30^, and lower Children’s Global Assessment Scale scores indicating greater functional impairment^31^. Positive BAGs have been related to depression and functional impairment^32^, psychosis, obsessive-compulsive symptoms, general psychopathology^33^, and a schizophrenia diagnosis^34^. Additionally, a higher BAG has been associated with psychosis risk in a sample of clinically high-risk youth^35^. Longitudinally, a deceleration in BAG was found for high familial risk adolescents who developed a mood disorder^36^, while a greater increase in BAG was found for adolescent females (but not males) with internalizing problems^37^.

The highlighted studies suggest that BAG carries prognostic information, positioning it as a putative early-warning tool. Clinical translation, however, demands more research to be carried out in addition to several safeguards. For example, BAG values could be interpreted against age- and sex-specific reference curves derived from large, harmonized cohorts—akin to the morphometric brain charts now available^38^. Reliability should generalize across scanners and pipelines, and incremental validity over established clinical and demographic predictors must be demonstrated in longitudinal cohorts. Until such benchmarks are met, BAG remains a promising research marker—useful for group-level risk stratification—rather than an individual-level clinical biomarker. The risk of misclassification and stigmatizing young people underlines this cautious stance.

### Physical and pubertal development

Studies consistently link BAG to pubertal development. Earlier pubertal timing, as measured via “puberty age,” is related to a higher BAG^39^. Additionally, higher parent-^8,16^ and youth-report^8^ pubertal development scale (PDS) scores have been related to an increased BAG, and annualized change in parent-report PDS has been related to annualized change in BAG^16^. Using a classifier trained to discriminate pre- versus post-menarche status, continuous menarche class probabilities have also been positively related to BAG^40^. Limited research examines BAG with other biological markers in youth. Preliminary evidence suggests BAG correlates with EpiAGE (an epigenetic aging measure)^41^, and both BAG and its change over time appear heritable^42^.

### Cognition

The relationship between BAG and cognition remains ambiguous, with studies reporting positive^31^, negative^43^, or no relationship^21^ between BAG and cognition during childhood and adolescence. Additionally, work has reported conflicting findings within the same study, related to different age ranges or models^8,44^. Notably, cognition measures vary widely, ranging from composite batteries (NIH Toolbox^8,21^ and Penn Computerized Neurocognitive Battery^45^), to specific tasks (e.g., Flanker Task^31^, working memory and numerical ability^46^). Mixed results have been found for IQ^43,47^, possibly due to differences in model features and samples.

### Environmental factors and life experiences

BAG has also been linked to a variety of environmental factors and life experiences, such as premature birth, socioeconomic status, and adversity. BAG appears to be higher in adolescents who are born very premature^47^. Longitudinally, neighborhood disadvantage in early adolescence is associated with a positive BAG, which decreases across adolescence^48^. In Cohen et al.^30^, a lower relative brain age (calculated using the residuals from regressing predicted age on chronological age) correlated with lower parental occupational prestige, lower public assistance enrollment, and more parent psychiatric diagnoses (but not parental education or income-to-needs ratio). An older BAG was likewise associated with environmental adversity; a composite score of multiple socioeconomic and adverse experience variables^32^. In an emotion-circuitry model, childhood abuse was linked to a lower BAG^49^. Dimensions of adversity have also been differentially related to BAG, such that a lower BAG is associated with factors related to emotional neglect, and an older BAG is associated with caregiver psychopathology, trauma exposure, family aggression, substance use, separation from biological parent, and socioeconomic disadvantage and neighborhood safety^23^.

With more brain-age prediction studies emerging in youth and inconsistent findings across various phenotypes, standard practices warrant scrutiny. Below, we highlight key challenges researchers should consider in order to ensure responsible, thorough neuroscience research.

## Challenges

Over the past decade, brain-age prediction has been increasingly used in child and adolescent populations to assess brain developmental stages. However, interpreting these models—specifically the estimated age and the BAG—remains challenging in young individuals, where effects of genetic and early-life factors are already observable^42,50^, and may mask the subtle effects of the variable of interest being investigated. There are also a number of methodological considerations, including universal challenges that may be more pronounced in youth, including age bias, multi-site scanner corrections, sample size, and design limitations, as well as youth-specific challenges such as nonmonotonic trajectories of brain development. Below, we summarize key challenges researchers should consider when applying brain-age prediction to neurodevelopmental samples.

### *Issue 1*: What does BAG represent in children and adolescents?

The BAG is defined as the difference between an individual’s predicted brain age and their chronological age. In this section, we use BAG as a generic shorthand for a brain-age deviation, whether expressed as the raw difference or as an age-corrected delta; the statistical distinctions and bias-correction procedures are treated in detail later, in *Issue 6*.

Existing research has not established what degree of variability in BAG is typical, and what may reflect substantially accelerated or decelerated development, meaning more work is needed to quantify the stability of BAG estimates over time and what factors underlie individual brain-age estimates^21^. Additionally, research has not yet determined whether BAGs persist across childhood and adolescence, or how common it is for someone to exhibit a BAG that narrows/converges with increasing age. Because this period is characterized by variable, nonlinear brain development^51^, and BAG condenses thousands of features into one global summary score, regional or mode-specific aging signals may overlook important regional nuances^52^. For instance, subcortical structures (e.g., amygdala, nucleus accumbens) often mature earlier than the prefrontal cortex^53^. This asynchrony can result in a developing brain that may appear “on time” globally yet harbor simultaneously delayed and accelerated tissue-specific trajectories. For example, multidimensional or tissue-specific clocks (e.g., mode-specific BAG, regional white-matter age) have revealed genetic associations invisible to a global score^52,54^. Furthermore, most models act as “black boxes,” obscuring which features contribute to model predictions^55,56^. These features may also vary over time and across individuals in terms of weight of contribution^57^.

In developing brains, deviations from the average may not signify pathology but could reflect normal variability, especially considering the high level of individual variance during childhood and adolescence^51^. For example, in adult samples, it is largely accepted that physical activity^58^, cardiometabolic risk factors^12,13^, and other environmental factors such as socioeconomic status and education^46,59^, influence BAG, with these influences accumulating over time. Because adult brain trajectories (e.g., increase in DTI FA, decrease in DTI MD, cortical thickness, and surface area^38,60,61^) are better established, interpreting BAG in adulthood is relatively more straightforward.

In contrast, youth studies are often plagued by narrow age ranges and nonmonotonic brain patterns (e.g., cortical surface area increases until ~10–11 years, then declines^62,63^), where it is likely that (1) negative lifestyle factors may not yet manifest as atypical brain development, and (2) quadratic/curvilinear effects may be hard for models to interpret. Moreover, some research has demonstrated the differential impact of factors related to emotional neglect being associated with delayed maturation, while other factors, such as parental psychopathology and disadvantageous SES to be associated with accelerated maturation^23^, meaning that in individuals experiencing co-occurring factors, this might reveal no deviation from typical development despite a larger net sum of a harsher environment.

Narrow age ranges in youth samples also mean BAGs in youth typically reflect weeks or months. How viable it is to look at this error score in the context of one particular measure that is meant to account for much of this explained variance should involve a level of skepticism. Further, a positive or negative BAG should not be equated with a direct acceleration or delay of the underlying biological maturation curve. Rather, BAG should be best regarded as a summary deviation—a proxy that aggregates many influences (sampling error, technical variance, lifestyle, genetic liability) into a single score. While such deviations have shown to be informative for brain-health phenotypes, attributing them to altered maturational processes or a causal gauge for developmental tempo will require more longitudinal modeling^55,64^ (see *Issue 3* for further discussion). Lastly, many adult studies differentiate between healthy and disorder-specific populations. Youth samples have the added layer of these studies potentially being carried out before the onset of clinical diagnoses for some of these individuals.

### Recommendation

Interpretation of BAG should be done within the context of normative developmental variations, i.e., recognizing that small deviations may fall within the range of typical developmental variability^65^. Considering confidence intervals and effect sizes rather than relying solely on point estimates may be better suited to convey the practical significance of between-group differences in BAG. Researchers should also be cautious when attributing clinical significance to minor deviations and should consider longitudinal assessments to observe changes over time, including nonlinear change in BAG tempo.

Additionally, responsible and precise language should be used when describing and interpreting the results of BAG-focused analyses. Specifically, it may be prudent to avoid the use of language such as “accelerated” and “decelerated” maturation when it is not yet clear whether BAG reflects ongoing maturational processes during childhood and adolescence. Instead, more neutral and precise terms could be beneficial, such as “older/younger appearing brain” or “positive/negative brain age gap”.

With mode-specific analyses uncovering 34 genetically informed aging axes in adults^52^, adapting such multi-axis clocks to youth could expose tissue- or network-specific maturational lags that a single BAG obscures. Here, regional brain-age models could be a promising avenue. While a number of recent studies in adult populations have applied regional brain-age models, this approach has been infrequently used in youth populations^49^. If regional brain-age prediction is not feasible, researchers may consider specifying the features used in the model and providing each feature’s contribution. See Ball et al.^66^ for an example of region contribution extracted from the Manifold structure for tissue volume.

Interpreting feature contributions can be useful for understanding the model in the context of known developmental changes, or relationships to the variables of interest, despite weight maps being complicated^67^. Tools like vip (variable importance plots) and SHAP (Shapley additive explanations) can reveal feature importance even in complex models^68^. SHAP offers a model-agnostic framework for evaluating feature influence in linear, nonlinear, and deep-learning approaches^69^. Methodological recommendations for the nonlinearity of youth brain development are addressed in *Issue 5*.

### *Issue 2*: Model training and sample choice

Model training and validation are critical for robust brain-age prediction. Models trained on data that do not represent the target population may lead to domain mismatch^70^, such as using adult-trained models on youth samples. Therefore, training on youth-specific data or including a substantial number of youth participants in the training set is important. The choice of modeling technique also matters^71^. Models may differ in key ways, such as their ability to extrapolate predictions beyond the sample observed in training. Tree-based algorithms such as random forests yield predictions confined to the observed training range^71^, whereas parametric or kernel-based methods can mathematically extrapolate—albeit often with high uncertainty—beyond that range.

Sample size is likewise critical^55^. Smaller datasets (and consequently lower power)^72^ are particularly problematic for neurodevelopmental studies, where inter-individual variability is high and thousands of participants may be required for robust brain-wide associations^73^. In comparison to traditional regression approaches, machine learning methods such as brain age approaches have two samples: a training set and a test set. Both sets must be sufficiently large, and different minimum sample sizes are potentially needed for each set, i.e., for model training versus model application and testing.

An additional consideration is whether models should be trained with sex-specificity in mind due to brain development variations in sex and pubertal development^42^. For instance, male youth exhibit more variability in brain structure than female youth^74^, and pubertal timing can influence brain development^75^, independently of chronological age. Brain-age models are able to robustly classify male and female brains^66^ despite small mean differences and neuroanatomical overlap. Research has reported 81% accuracy in sex prediction^66^, with higher BAG in female youth^21^, likely reflecting accelerated maturation in mid-to-late adolescence^42^. Research indicates about a 1-year difference at ages 14–16, with some convergence at 18 years of age, with males catching up to females^42^. This highlights the importance of accounting for sex and puberty during critical developmental periods.

### Recommendations

First, ensure the training data reflects the target population to capture unique developmental patterns. The Brain Age Standardized Evaluation (BASE) provides a framework for evaluating model training and robust performance assessment^76^.

Second, use adequately large training and testing samples. Smaller datasets often fail to capture the high inter-individual variability in youth. Empirical learning curves show that Mean Absolute Error (MAE)—defined as the average of the absolute differences between each person’s predicted brain age and their actual chronological age—plateaus at roughly 20 high-quality, well-controlled scans per 6-month age bin. This equates to about 250–300 participants across a typical 6-year (11–17 years) window, with only marginal gains in accuracy after^48,77^. However, this is a practical minimum for studies using atlas-level features and classical regressors. We recommend (i) plotting a learning curve to confirm where your plateau lies and (ii) treating these numbers as starting points rather than hard cut-offs. Larger cohorts (>500) can still boost cross-scanner and cross-ethnic generalizability and provide the statistical power needed for smaller developmental differences. Multi-site harmonization, transfer-learning, and normative modeling all benefit from larger cohorts even when MAE has leveled off. Moreover, pooling multiple datasets and using cross-validation (e.g., k-fold or leave-one-out) can mitigate overfitting and yield reliable estimates. If a dataset is limited, applying a pre-trained model may be preferable to training a new one on insufficient data. Ideally, the training set should include data from a heterogeneous variety of scanners, as this helps generalization to external samples^56^.

Finally, consider stratifying models by sex or pubertal status to account for biological variability in development. Covering the entire span of puberty is especially helpful for capturing critical developmental trends.

### *Issue 3*: Design

The current literature is limited by a reliance on cross-sectional designs and limited reproducibility. While cross-sectional studies can provide valuable snapshots of developmental differences, they are insufficient for testing hypotheses about the speed, timing, or trajectory of brain development^78^. This undermines claims of delayed or accelerated maturation during this highly variable and nonlinear period.

Longitudinal designs are essential for distinguishing between the speed and timing of maturation, clarifying to what extent variations in BAG reflect true deviations (e.g., accelerated or decelerated development). Cross-sectional estimates risk conflating group differences with developmental differences, as they cannot account for individual variability in brain development over time. This is particularly problematic in childhood and adolescence, when rapid, heterogeneous changes occur^2,63^. Longitudinal designs are uniquely positioned to identify sensitive periods or turning points in brain age trajectories, shedding light on whether deviations in BAG are transient or stable indicators of risk.

Recent work has quantified the extensive nature of individual variation in brain development during childhood and adolescence, illustrating the difficulty of differentiating altered developmental trajectories from normative variation^51^. Longitudinal data is also an avenue to explore the impact of not only single time point estimates, but also how changes in BAG may relate to different outcomes, and how these relationships can shift across development. For example, Rakesh et al.^48^ linked neighborhood disadvantage to a positive BAG in early adolescence, and a deceleration in BAG in later adolescence, suggesting timing-dependent effects. Though a cross-sectional BAG might indicate persistent risk, more longitudinal work is needed to confirm when BAG truly reflects accelerated or delayed maturation and how it relates to health concerns.

### Recommendations

Longitudinal data is essential to address challenges in design and developmental variability^79^. By conducting longitudinal brain age studies, we can better differentiate between normative variation and altered developmental pathways, resulting in a clearer understanding of BAG and true maturational speed. This is particularly important when brain age metrics are coupled with youth clinical or behavioral assessments, where claims of atypical brain development may arise. Tracking the same individuals over time may reveal whether BAG deviations signify genuine acceleration or delay in maturation.

### *Issue 4*: Model performance metrics

Model performance metrics, such as MAE and root mean square error (RMSE), are central to evaluating brain-age models but can be difficult to interpret across different studies and age ranges^80^, never mind developmental stages. In youth samples, MAEs are typically much lower (e.g., 0.5–1.5 years)^19,55^ than in adult populations, where values of 3–6 years are considered good performance^55,56^. However, with MAE being scale-dependent^81^, these raw metrics can be misleading without context. For example, an MAE of 0.35 years in a youth sample may appear to outperform models with MAEs of 3.5 years in adult samples. However, both represent an approximate deviation of 7% of their total age ranges (9–14 and 40–87, respectively). It remains a task of future research to determine how we compare these error and performance metrics across youth versus adult samples.

Relatedly, MAE and similar metrics are inherently influenced by the age range of the training and test samples^80^. Wider age ranges tend to increase prediction errors because they introduce more variability in brain structure and function. Conversely, narrower ranges, especially during periods of rapid anatomical change, can yield artificially low MAEs and *r* values that may not generalize to other contexts^66,80^. These findings underscore the importance of interpreting performance metrics in the context of age range, developmental stage, and variability.

### Recommendations

To improve the interpretability and comparability of performance metrics across studies, researchers should consider reporting the MAE together with the chronological-age range of the test set and, where cross-study comparison is a goal, optionally add a normalized figure. This supplementary value contextualizes performance while providing a context-sensitive comparison. For example, providing MAE/RMSE for absolute error and the cross-validated predictive *R*^2^—the proportion of age variance explained in each held-out fold—listed fold-by-fold rather than as a single mean, in line with BASE^76^ and BabyPy^82^ guidelines. Developing shared reference datasets and benchmarking frameworks would further standardize practice and harmonize reporting—an especially important goal given the scarcity of distinct youth cohorts, which currently restricts opportunities for truly independent model evaluation.

### *Issue 5*: Nonlinearity

Nonmonotonic and nonlinear brain patterns are especially pertinent during childhood and adolescence. While there is a growing expectation that nonlinear and ensemble algorithms (e.g., kernel methods, deep learning) will better capture these complexities, evidence shows that such methods do not necessarily outperform simpler linear models in practice^83^. In fact, research shows that regularized linear algorithms are as effective as nonlinear and ensemble algorithms, while significantly reducing computational costs^84^. A key factor is that neurodevelopmental datasets—often constrained by modest sample sizes and measurement noise—may not have sufficiently robust nonlinear signals for complex models to exploit, leaving linear approaches performing comparably well.

Moreover, deep convolutional architectures assume translation invariance and compositional structure, assumptions that may not readily apply to the fixed anatomical organization of the human brain. Schulz and colleagues^83^ demonstrate that when you artificially inject high levels of noise into a dataset, kernel and deep models eventually perform no better than linear models due to the noise washing out the higher-order patterns. Even when genuine nonlinear trajectories exist, the interpretability of black-box algorithms remains challenging. As we assume the existence of BAG in the age prediction model, a good predictive model for brain age estimates should not overfit the data and yield a perfect prediction for chronological age^85^, as that yields no meaningful variance in the BAG measure. Simpler methods grounded in known developmental principles can capture a large portion of the variance without risking overfitting, especially when samples are small or noisy.

### Recommendations

Making recommendations for the challenge of nonlinearity is difficult. Ideally, we should consider nonlinear modeling techniques to better capture complex developmental trajectories and asynchronicities. Such models may help account for the dynamic and regionally specific growth spurts or regressive processes (e.g., pruning) that define childhood and adolescence^6^. Machine learning techniques capable of handling nonlinear effects, such as Gaussian Process Regression, XGBoost, and Support Vector Regression that use nonlinear mapping functions (i.e., kernels) to discover boundaries in the data by creating an implicit feature space^86^, or neural networks with appropriate regularization, may be better suited for predicting brain age in youth samples. Where sample size allows, researchers may also deploy multi-axis or modality-specific brain-age clocks that partition nonlinear maturation into distinct aging trajectories (see “Conclusion” for more). However, at current data scales and quality, researchers may find that linear or simpler nonlinear strategies provide a more transparent and practical starting point. Here, it may be more important to avoid overfitting by, e.g., imposing a higher level regularization^87^ and using lower-dimensional linear models. If you suspect strong nonlinear effects (e.g., quadratic or cubic age trends) and have enough participants spanning the age range of interest, you may consider kernel or neural-network approaches only after simpler spline or polynomial approaches (or well-powered linear models) have been tested. As sample sizes grow and noise reduction techniques improve, however, advanced nonlinear models may eventually prove valuable for elucidating subtle developmental irregularities that simpler approaches might overlook.

### *Issue 6*: Corrections and other biases

Brain-age models face biases that can impact their accuracy, interpretability, and generalizability. These include age dependence, which can be addressed through bias correction^80^ as well as batch effects from multi-site MRI datasets, which can be mitigated through harmonization techniques^88,89^.

The most widely reported index—BAG—is a raw difference between an individual’s predicted and chronological age. Because this is algebraically proportional to the out-of-sample prediction error, it is *necessarily* correlated with age, leading to systematic overestimation in younger participants and underestimation in older ones. Smith and colleagues^90^ showed that, in the extreme case where imaging features carry no true age signal, BAG collapses to a simple linear function of chronological age, so any downstream association with cognition, psychopathology, or environmental risks being a proxy for residual age effects. To mitigate this “regression-to-the-mean” bias, several corrected variants are now common, such as regressing out age effects from model predictions, including chronological age as a covariate in analyses, and correcting predictions using slope and intercept adjustments^91,92^. While these methods can reduce bias, they are not without trade-offs. Certain correction techniques, particularly those based on regression adjustments, can artificially inflate model performance metrics such as *R*^2^ and reduce error measures^93^.

Multi-site datasets, such as the Adolescent Brain and Cognitive Development Study^94^ and IMAGEN^95^, are invaluable for training and testing brain-age models. However, these datasets are often subject to systematic differences introduced by varying imaging sites and scanner protocols^96^. Without correction, scanner/site effects inflate apparent inter-individual variance and can bias BAG estimates if not addressed. Several methods have been proposed to address site and scanner effects, for example, including site/scanner as a covariate in statistical analyses. Alternatively, one can utilize a suite of harmonization tools such as NeuroHarmonize, CovBat, RAVEL, cross-sectional- and longitudinal ComBat, which have been shown to reduce scanner-induced variability effectively^88,97^. These approaches reduce feature-level variance attributable to technical artifacts, although recent work^98^ shows that such corrections do not invariably improve brain-age prediction accuracy. Despite their utility, harmonization techniques must be applied cautiously to avoid data leakage^99^.

### Recommendations

With no consensus on the best correction method, it is advisable for researchers to assess the degree of bias in raw predictions before applying corrections and report both corrected and uncorrected metrics, as recommended by de Lange et al.^80^, and visualize residuals across the age span to provide transparency and enable meaningful comparisons.

When addressing multi-site effects, harmonization techniques can be particularly useful for reducing variability in brain measures due to technical artifacts. Harmonization parameters should be learned only on the training data within each cross-validation fold and then applied unchanged to the held-out test set. Estimating them on the full dataset before the split leaks information from test to train and can inflate performance, whereas re-estimating them separately on the test set avoids leakage but puts train and test features on different scales, undermining comparability. Well-designed pipelines, therefore, fit the correction in the training partition and apply that fixed transformation to the test partition, ensuring bias removal without overfitting. Segmentation routines, such as using standard adult reference data^100^ can also introduce systemic bias. Franke and colleagues^101^ avoided this issue by using the Template-O-Matic toolbox^102^, which generates a sample-specific template where tissue segmentation does not rely on prior information maps but rather solely on voxel intensity. Deep learning methods are also a promising avenue here, using data-driven representations of various global and local data features and removing the reliance on data preprocessing to extract meaningful features^55^ (Table 1).

**Table 1:** Issues and recommendations

1. BAG interpretation	Use longitudinal data to capture BAG stability and dynamics
2. Model training	Ensure diverse, representative samples with sufficient power
3. Study design	Prioritize longitudinal designs
4. Model performance and specificity	Report multiple fit metrics for comparability and weight/features used
5. Nonlinearity	Address nonmonotonic and nonlinear trends
6. Corrections and other biases	Address site/scanner effects and adjust for biases

## Conclusion

Our work highlights the potential for refining the use of the brain age framework in developmental samples. As an exciting frontier in child and adolescent neurodevelopmental research, brain-age prediction offers a powerful way to capture the unique and dynamic changes occurring during these critical periods. Yet, challenges such as how to interpret BAG given the nonmonotonic and nonlinear brain patterns in youth, model training and sample size, lack of longitudinal datasets, insufficient reporting of multiple model performance metrics, and other biases such as site and scanner variability are emblematic of broader methodological issues in developmental neuroscience. Addressing these challenges, alongside others discussed in this Perspective paper, is crucial not only for improving methodologies but also for ensuring that these models yield meaningful insights about the developing brain.

Moving forward, the field would benefit from establishing standard best practices for applying brain-age prediction in youth populations and improving efforts that foster reproducibility and cross-study integration in brain age research. Progress may also come from **e**xpanding brain-age prediction beyond a single chronological clock. Data-driven analyses in adults reveal multiple orthogonal aging axes^52^ and tissue-specific or multimodal models such as BrainAgeNeXt^103^ detect white-matter and cross-modal signals that a global score misses. Tailoring these multi-axis frameworks to youth cohorts may offer a more nuanced, biologically specific picture of neurodevelopmental timing and tempo. Lastly, we also encourage open science practices, including pre-registering studies, sharing model code and weight maps, and providing detailed methodology. This would facilitate replication, cross-sample validation, and continued innovation in youth brain-age prediction.

## Supplementary information

Supplementary Infomation

## References

[1] Blakemore, S.-J. Imaging brain development: the adolescent brain. *NeuroImage* **61**, 397–406 (2012). 22178817 10.1016/j.neuroimage.2011.11.080

[2] Mills, K. L. et al. Structural brain development between childhood and adulthood: convergence across four longitudinal samples. *NeuroImage* **141**, 273–281 (2016). 27453157 10.1016/j.neuroimage.2016.07.044 PMC5035135

[3] Mills, K. L. & Tamnes, C. K. Longitudinal structural and functional brain development in adolescence. in *The Oxford Handbook of Developmental Cognitive Neuroscience* 75–98 (Oxford University Press, 2022).

[4] Lebel, C. & Deoni, S. The development of brain white matter microstructure. *NeuroImage* **182**, 207–218 (2018). 29305910 10.1016/j.neuroimage.2017.12.097 PMC6030512

[5] Rosenberg, B. M., Mennigen, E., Monti, M. M. & Kaiser, R. H. Functional segregation of human brain networks across the lifespan: an exploratory analysis of static and dynamic resting-state functional connectivity. *Front. Neurosci*. **14**, 561594 (2020). 10.3389/fnins.2020.561594 PMC7752769 33363450

[6] Norbom, L. B. et al. New insights into the dynamic development of the cerebral cortex in childhood and adolescence: integrating macro- and microstructural MRI findings. *Prog. Neurobiol.* **204**, 102109 (2021). 34147583 10.1016/j.pneurobio.2021.102109

[7] Franke, K., Ziegler, G., Klöppel, S. & Gaser, C. Estimating the age of healthy subjects from T1-weighted MRI scans using kernel methods: exploring the influence of various parameters. *NeuroImage* **50**, 883–892 (2010). 20070949 10.1016/j.neuroimage.2010.01.005

[8] Whitmore, L. B., Weston, S. J. & Mills, K. L. BrainAGE as a measure of maturation during early adolescence. *Imaging Neurosci.* **1**, 1–21 (2023). 10.1162/imag_a_00037 PMC12007541 40799695

[9] Elliott, M. L. et al. Brain-age in midlife is associated with accelerated biological aging and cognitive decline in a longitudinal birth cohort. *Mol. Psychiatry* **26**, 3829–3838 (2021). 31822815 10.1038/s41380-019-0626-7 PMC7282987

[10] Seitz-Holland, J., Haas, S. S., Penzel, N., Reichenberg, A. & Pasternak, O. BrainAGE, brain health, and mental disorders: a systematic review. *Neurosci. Biobehav. Rev.* **159**, 105581 (2024). 38354871 10.1016/j.neubiorev.2024.105581 PMC11119273

[11] Anatürk, M. et al. Prediction of brain age and cognitive age: quantifying brain and cognitive maintenance in aging. *Hum. Brain Mapp.* **42**, 1626–1640 (2021). 33314530 10.1002/hbm.25316 PMC7978127

[12] Beck, D. et al. Adipose tissue distribution from body MRI is associated with cross-sectional and longitudinal brain age in adults. *NeuroImage Clin.* **33**, 102949 (2022). 35114636 10.1016/j.nicl.2022.102949 PMC8814666

[13] Beck, D. et al. Cardiometabolic risk factors associated with brain age and accelerate brain ageing. *Hum. Brain Mapp.* **43**, 700–720 (2022). 34626047 10.1002/hbm.25680 PMC8720200

[14] Beck, D. et al. Dissecting unique and common variance across body and brain health indicators using age prediction. *Hum. Brain Mapp.* **45**, e26685 (2024). 38647042 10.1002/hbm.26685 PMC11034003

[15] de Lange, A.-M. G. et al. Multimodal brain-age prediction and cardiovascular risk: The Whitehall II MRI sub-study. *NeuroImage* **222**, 117292 (2020). 32835819 10.1016/j.neuroimage.2020.117292 PMC8121758

[16] Holm, M. C. et al. Linking brain maturation and puberty during early adolescence using longitudinal brain age prediction in the ABCD cohort. *Dev. Cogn. Neurosci.* **60**, 101220 (2023). 36841180 10.1016/j.dcn.2023.101220 PMC9972398

[17] Kaufmann, T. et al. Common brain disorders are associated with heritable patterns of apparent aging of the brain. *Nat. Neurosci.* **22**, 1617–1623 (2019). 31551603 10.1038/s41593-019-0471-7 PMC6823048

[18] Tønnesen, S. et al. Brain age prediction reveals aberrant brain white matter in schizophrenia and bipolar disorder: a multisample diffusion tensor imaging study. *Biol. Psychiatry Cogn. Neurosci. Neuroimaging* **5**, 1095–1103 (2020). 32859549 10.1016/j.bpsc.2020.06.014

[19] Franke, K. & Gaser, C. Longitudinal changes in individual BrainAGE in healthy aging, mild cognitive impairment, and Alzheimer’s disease. *GeroPsych J. Gerontopsychol. Geriatr. Psychiatry* **25**, 235–245 (2012).

[20] Subramaniapillai, S. et al. Sex differences in brain aging among adults with family history of Alzheimer’s disease and APOE4 genetic risk. *NeuroImage Clin.* **30**, 102620 (2021). 33857772 10.1016/j.nicl.2021.102620 PMC8065341

[21] Ball, G., Kelly, C. E., Beare, R. & Seal, M. L. Individual variation underlying brain age estimates in typical development. *NeuroImage* **235**, 118036 (2021). 33838267 10.1016/j.neuroimage.2021.118036

[22] Han, L. K. M. et al. Brain aging in major depressive disorder: results from the ENIGMA major depressive disorder working group. *Mol. Psychiatry* **26**, 5124–5139 (2020). 32424236 10.1038/s41380-020-0754-0 PMC8589647

[23] Beck, D. et al. Dimensions of early-life adversity are differentially associated with patterns of delayed and accelerated brain maturation. *Biol. Psychiatry* **97**, 64–72 (2024). 39084501 10.1016/j.biopsych.2024.07.019

[24] American Academy of Pediatrics. *Bright Futures: Guidelines for Health Supervision of Infants, Children, and Adolescents* 4th edn. https://www.scribd.com/document/410609912/Bright-Futures-Guidelines-for-Health-Supervision-of-Infants-Children-and-Adolescents-4TH-pdf (American Academy of Pediatrics, 2017).

[25] Centers for Disease Control and Prevention (CDC). Positive parenting tips: preschoolers (3–5 years). https://www.cdc.gov/child-development/positive-parenting-tips/preschooler-3-5-years.html (CDC, 2024).

[26] World Health Organization (WHO). Adolescent health. https://www.who.int/health-topics/adolescent-health (WHO, 2025).

[27] Zhou, Z. et al. Differential effects of generalized anxiety and separation anxiety on brain structural development during adolescence. *J. Affect. Disord.* **339**, 478–485 (2023). 37442456 10.1016/j.jad.2023.07.056

[28] Tunç, B. et al. Deviation from normative brain development is associated with symptom severity in autism spectrum disorder. *Mol. Autism* **10**, 46 (2019). 31867092 10.1186/s13229-019-0301-5 PMC6907209

[29] Kurth, F. et al. Preliminary evidence for a lower brain age in children with attention-deficit/hyperactivity disorder. *Front. Psychiatry* **13**, 1019546 (2022). 10.3389/fpsyt.2022.1019546 PMC9755736 36532197

[30] Cohen, J. W. et al. Relative brain age is associated with socioeconomic status and anxiety/depression problems in youth. *Dev. Psychol.* **60**, 199–209 (2024). 37747510 10.1037/dev0001593 PMC10993304

[31] Luna, A. et al. Maturity of gray matter structures and white matter connectomes, and their relationship with psychiatric symptoms in youth. *Hum. Brain Mapp.* **42**, 4568–4579 (2021). 34240783 10.1002/hbm.25565 PMC8410534

[32] Drobinin, V. et al. The developmental brain age is associated with adversity, depression, and functional outcomes among adolescents. *Biol. Psychiatry Cogn. Neurosci. Neuroimaging* **7**, 406–414 (2022). 34555562 10.1016/j.bpsc.2021.09.004

[33] Cropley, V. L. et al. Brain-predicted age associates with psychopathology dimensions in youths. *Biol. Psychiatry Cogn. Neurosci. Neuroimaging* **6**, 410–419 (2021). 32981878 10.1016/j.bpsc.2020.07.014

[34] Truelove-Hill, M. et al. A multidimensional neural maturation index reveals reproducible developmental patterns in children and adolescents. *J. Neurosci.* **40**, 1265–1275 (2020). 31896669 10.1523/JNEUROSCI.2092-19.2019 PMC7002145

[35] Chung, Y. et al. Use of machine learning to determine deviance in neuroanatomical maturity associated with future psychosis in youths at clinically high risk. *JAMA Psychiatry* **75**, 960–968 (2018). 29971330 10.1001/jamapsychiatry.2018.1543 PMC6142910

[36] de Nooij, L. et al. Longitudinal trajectories of brain age in young individuals at familial risk of mood disorder from the Scottish Bipolar Family Study. *Wellcome Open Res.* **4**, 206 (2020). 32954013 10.12688/wellcomeopenres.15617.1 PMC7479500

[37] MacSweeney, N. et al. Multimodal brain age indicators of internalizing problems in early adolescence: a longitudinal investigation. *Biol. Psychiatry Cogn. Neurosci. Neuroimaging* **10**, 475–484 (2024). 39566883 10.1016/j.bpsc.2024.11.003

[38] Bethlehem, R. A. I. et al. Brain charts for the human lifespan. *Nature* **604**, 525–533 (2022). 35388223 10.1038/s41586-022-04554-y PMC9021021

[39] Dehestani, N., Whittle, S., Vijayakumar, N. & Silk, T. J. Developmental brain changes during puberty and associations with mental health problems. *Dev. Cogn. Neurosci.* **60**, 101227 (2023). 36933272 10.1016/j.dcn.2023.101227 PMC10036507

[40] Gottschewsky, N., Kraft, D. & Kaufmann, T. Developmental brain changes during puberty and associations with mental health problems. *Biol. Sex Differ.* **15**, 25 (2024).38532493

[41] Mareckova, K. et al. Longitudinal study of epigenetic aging and its relationship with brain aging and cognitive skills in young adulthood. *Front. Aging Neurosci.* **15**, 1215957 (2023). 37593374 10.3389/fnagi.2023.1215957 PMC10427722

[42] Brouwer, R. M. et al. The speed of development of adolescent brain age depends on sex and is genetically determined. *Cereb. Cortex* **31**, 1296–1306 (2021). 33073292 10.1093/cercor/bhaa296 PMC8204942

[43] Lewis, J. D., Evans, A. C. & Tohka, J. T1 white/gray contrast as a predictor of chronological age, and an index of cognitive performance. *NeuroImage* **173**, 341–350 (2018). 29501876 10.1016/j.neuroimage.2018.02.050

[44] Ullman, H. & Klingberg, T. Timing of white matter development determines cognitive abilities at school entry but not in late adolescence. *Cereb. Cortex* **27**, 4516–4522 (2017). 27550867 10.1093/cercor/bhw256

[45] Erus, G. et al. Imaging patterns of brain development and their relationship to cognition. *Cereb. Cortex* **25**, 1676–1684 (2015). 24421175 10.1093/cercor/bht425 PMC4428302

[46] Steffener, J. et al. Differences between chronological and brain age are related to education and self-reported physical activity. *Neurobiol. Aging* **40**, 138 (2016). 26973113 10.1016/j.neurobiolaging.2016.01.014 PMC4792330

[47] Hedderich, D. M. et al. Increased brain age gap estimate (BrainAGE) in young adults after premature birth. *Front. Aging Neurosci*. **13**, 653365 (2021). 10.3389/fnagi.2021.653365 PMC8047054 33867970

[48] Rakesh, D. et al. Neighborhood disadvantage and longitudinal brain-predicted-age trajectory during adolescence. *Dev. Cogn. Neurosci.* **51**, 101002 (2021). 34411954 10.1016/j.dcn.2021.101002 PMC8377545

[49] Keding, T. J. et al. Differential patterns of delayed emotion circuit maturation in abused girls with and without internalizing psychopathology. *Am. J. Psychiatry* **178**, 1026–1036 (2021). 34407623 10.1176/appi.ajp.2021.20081192 PMC8570983

[50] Vidal-Pineiro, D. et al. Individual variations in ‘brain age’ relate to early-life factors more than to longitudinal brain change. *eLife* **10**, e69995 (2021). 34756163 10.7554/eLife.69995 PMC8580481

[51] Mills, K. L. et al. Inter-individual variability in structural brain development from late childhood to young adulthood. *NeuroImage* **242**, 118450 (2021). 34358656 10.1016/j.neuroimage.2021.118450 PMC8489572

[52] Smith, S. M. et al. Brain aging comprises many modes of structural and functional change with distinct genetic and biophysical associations. *eLife* **9**, e52677 (2020). 32134384 10.7554/eLife.52677 PMC7162660

[53] Mills, K. L., Goddings, A.-L., Clasen, L. S., Giedd, J. N. & Blakemore, S.-J. The developmental mismatch in structural brain maturation during adolescence. *Dev. Neurosci.* **36**, 147–160 (2014). 24993606 10.1159/000362328

[54] Lee, P.-L. et al. Regional rather than global brain age mediates cognitive function in cerebral small vessel disease. *Brain Commun.* **4**, fcac233 (2022). 36196084 10.1093/braincomms/fcac233 PMC9525017

[55] Cole, J. & Franke, K. Predicting age using neuroimaging: innovative brain ageing biomarkers. *Trends Neurosci.* **40**, 681–690 (2017). 29074032 10.1016/j.tins.2017.10.001

[56] Liem, F. et al. Predicting brain-age from multimodal imaging data captures cognitive impairment. *NeuroImage* **148**, 179–188 (2017). 27890805 10.1016/j.neuroimage.2016.11.005

[57] Brown, T. T. et al. Neuroanatomical assessment of biological maturity. *Curr. Biol.* **22**, 1693–1698 (2012). 22902750 10.1016/j.cub.2012.07.002 PMC3461087

[58] Sanders, A.-M. et al. Linking objective measures of physical activity and capability with brain structure in healthy community dwelling older adults. *NeuroImage Clin.* **31**, 102767 (2021). 34330086 10.1016/j.nicl.2021.102767 PMC8329542

[59] Busby, N. et al. Lower socioeconomic status is associated with premature brain aging. *Neurobiol. Aging* **130**, 135–140 (2023). 37506551 10.1016/j.neurobiolaging.2023.06.012 PMC13277732

[60] Beck, D. et al. White matter microstructure across the adult lifespan: a mixed longitudinal and cross-sectional study using advanced diffusion models and brain-age prediction. *NeuroImage* **224**, 117441 (2021). 33039618 10.1016/j.neuroimage.2020.117441

[61] Westlye, L. T. et al. Life-span changes of the human brain white matter: diffusion tensor imaging (DTI) and volumetry. *Cereb. Cortex* **20**, 2055–2068 (2010). 20032062 10.1093/cercor/bhp280

[62] Raznahan, A. et al. How does your cortex grow? *J. Neurosci.* **31**, 7174–7177 (2011). 21562281 10.1523/JNEUROSCI.0054-11.2011 PMC3157294

[63] Wierenga, L. M., Langen, M., Oranje, B. & Durston, S. Unique developmental trajectories of cortical thickness and surface area. *NeuroImage* **87**, 120–126 (2014). 24246495 10.1016/j.neuroimage.2013.11.010

[64] Liang, H., Zhang, F. & Niu, X. Investigating systematic bias in brain age estimation with application to post-traumatic stress disorders. *Hum. Brain Mapp.* **40**, 3143–3152 (2019). 30924225 10.1002/hbm.24588 PMC6865701

[65] Lenroot, R. K. & Giedd, J. N. Brain development in children and adolescents: insights from anatomical magnetic resonance imaging. *Neurosci. Biobehav. Rev.* **30**, 718–729 (2006). 16887188 10.1016/j.neubiorev.2006.06.001

[66] Ball, G., Adamson, C., Beare, R. & Seal, M. L. Modelling neuroanatomical variation during childhood and adolescence with neighbourhood-preserving embedding. *Sci. Rep.* **7**, 17796 (2017). 29259302 10.1038/s41598-017-18253-6 PMC5736651

[67] Haufe, S. et al. On the interpretation of weight vectors of linear models in multivariate neuroimaging. *NeuroImage* **87**, 96–110 (2014). 24239590 10.1016/j.neuroimage.2013.10.067

[68] Greenwell, B. M. & Boehmke, B. C. Variable importance plots—an introduction to the vip Package. *R. J.* **12**, 343 (2020).

[69] Lundberg, S. M. et al. From local explanations to global understanding with explainable AI for trees. *Nat. Mach. Intell.* **2**, 56–67 (2020). 32607472 10.1038/s42256-019-0138-9 PMC7326367

[70] Wood, D. A. et al. Optimising brain age estimation through transfer learning: a suite of pre-trained foundation models for improved performance and generalisability in a clinical setting. *Hum. Brain Mapp.* **45**, e26625 (2024). 38433665 10.1002/hbm.26625 PMC10910262

[71] Breiman, L. Random forests. *Mach. Learn.* **45**, 5–32 (2001).

[72] Button, K. S. et al. Power failure: Why small sample size undermines the reliability of neuroscience. *Nat. Rev. Neurosci.* **14**, 365–376 (2013). 23571845 10.1038/nrn3475

[73] Marek, S. et al. Reproducible brain-wide association studies require thousands of individuals. *Nature* **603**, 654–660 (2022). 35296861 10.1038/s41586-022-04492-9 PMC8991999

[74] Wierenga, L. M. et al. A key characteristic of sex differences in the developing brain: greater variability in brain structure of boys than girls. *Cereb. Cortex* **28**, 2741–2751 (2018). 28981610 10.1093/cercor/bhx154 PMC6041809

[75] Herting, M. M. & Sowell, E. R. Puberty and structural brain development in humans. *Front. Neuroendocrinol.* **44**, 122–137 (2017). 28007528 10.1016/j.yfrne.2016.12.003 PMC5612369

[76] Dular, L. & Špiclin, Ž.Alzheimer’s Disease Neuroimaging Initiative BASE: Brain Age Standardized Evaluation. *NeuroImage* **285**, 120469 (2024). 38065279 10.1016/j.neuroimage.2023.120469

[77] Griffiths-King, D., Wood, A. G. & Novak, J. Predicting ‘Brainage’ in late childhood to adolescence (6-17yrs) using structural MRI, morphometric similarity, and machine learning. *Sci. Rep.* **13**, 15591 (2023). 37730747 10.1038/s41598-023-42414-5 PMC10511546

[78] Kraemer, H. C., Yesavage, J. A., Taylor, J. L. & Kupfer, D. How can we learn about developmental processes from cross-sectional studies, or can we? *Am. J. Psychiatry* **157**, 163–171 (2000). 10671382 10.1176/appi.ajp.157.2.163

[79] Foulkes, L. & Blakemore, S.-J. Studying individual differences in human adolescent brain development. *Nat. Neurosci.* **21**, 315–323 (2018). 29403031 10.1038/s41593-018-0078-4

[80] de Lange, A.-M. G. et al. Mind the gap: performance metric evaluation in brain-age prediction. *Hum. Brain Mapp.* **43**, 3113–3129 (2022). 35312210 10.1002/hbm.25837 PMC9188975

[81] Hyndman, R. J. & Koehler, A. B. Another look at measures of forecast accuracy. *Int. J. Forecast.* **22**, 679–688 (2006).

[82] Biondo, F. et al. BabyPy: a brain-age model for infancy, childhood, and adolescence. Preprint at https://www.biorxiv.org/content/10.1101/2025.02.05.636598v2 (2025).

[83] Schulz, M.-A. et al. Different scaling of linear models and deep learning in UKBiobank brain images versus machine-learning datasets. *Nat. Commun.* **11**, 4238 (2020). 32843633 10.1038/s41467-020-18037-z PMC7447816

[84] Han, J., Kim, S. Y., Lee, J. & Lee, W. H. Brain age prediction: a comparison between machine learning models using brain morphometric data. *Sensors* **22**, 8077 (2022). 36298428 10.3390/s22208077 PMC9608785

[85] Niu, X., Taylor, A., Shinohara, R. T., Kounios, J. & Zhang, F. Multidimensional brain-age prediction reveals altered brain developmental trajectory in psychiatric disorders. *Cereb. Cortex* **32**, 5036–5049 (2022). 35094075 10.1093/cercor/bhab530

[86] Hofmann, T., Schölkopf, B. & Smola, A. J. Kernel methods in machine learning. *Ann. Stat.* **36**, 1171–1220 (2008).

[87] Bashyam, V. M. et al. MRI signatures of brain age and disease over the lifespan based on a deep brain network and 14 468 individuals worldwide. *Brain* **143**, 2312–2324 (2020). 32591831 10.1093/brain/awaa160 PMC7364766

[88] Beer, J. C. et al. Longitudinal ComBat: a method for harmonizing longitudinal multi-scanner imaging data. *NeuroImage* **220**, 117129 (2020). 32640273 10.1016/j.neuroimage.2020.117129 PMC7605103

[89] Lombardi, A. et al. Extensive evaluation of morphological statistical harmonization for brain age prediction. *Brain Sci.* **10**, 364 (2020). 32545374 10.3390/brainsci10060364 PMC7349402

[90] Smith, S. M., Vidaurre, D., Alfaro-Almagro, F., Nichols, T. E. & Miller, K. L. Estimation of brain age delta from brain imaging. *NeuroImage* **200**, 528–539 (2019). 31201988 10.1016/j.neuroimage.2019.06.017 PMC6711452

[91] de Lange, A.-M. G. & Cole, J. Commentary: Correction procedures in brain-age prediction. *NeuroImage Clin.* **26**, 102229 (2020). 32120292 10.1016/j.nicl.2020.102229 PMC7049655

[92] Beheshti, I., Nugent, S., Potvin, O. & Duchesne, S. Bias-adjustment in neuroimaging-based brain age frameworks: a robust scheme. *NeuroImage Clin.* **24**, 102063 (2019). 31795063 10.1016/j.nicl.2019.102063 PMC6861562

[93] Butler, E. R. et al. Pitfalls in brain age analyses. *Hum. Brain Mapp.* **42**, 4092–4101 (2021). 34190372 10.1002/hbm.25533 PMC8357007

[94] Casey, B. J. et al. The Adolescent Brain Cognitive Development (ABCD) study: imaging acquisition across 21 sites. *Dev. Cogn. Neurosci.* **32**, 43–54 (2018). 29567376 10.1016/j.dcn.2018.03.001 PMC5999559

[95] Mascarell-Maričić, L. et al. The IMAGEN study: a decade of imaging genetics in adolescents. *Mol. Psychiatry* **25**, 2648–2671 (2020). 32601453 10.1038/s41380-020-0822-5 PMC7577859

[96] Han, X. et al. Reliability of MRI-derived measurements of human cerebral cortical thickness: the effects of field strength, scanner upgrade and manufacturer. *NeuroImage* **32**, 180–194 (2006). 16651008 10.1016/j.neuroimage.2006.02.051

[97] Fortin, J.-P. et al. Harmonization of cortical thickness measurements across scanners and sites. *NeuroImage* **167**, 104–120 (2018). 29155184 10.1016/j.neuroimage.2017.11.024 PMC5845848

[98] Yu, Y. et al. Brain-age prediction: systematic evaluation of site effects, and sample age range and size. *Hum. Brain Mapp.* **45**, e26768 (2024). 38949537 10.1002/hbm.26768 PMC11215839

[99] Marzi, C. et al. Efficacy of MRI data harmonization in the age of machine learning: a multicenter study across 36 datasets. *Sci. Data* **11**, 115 (2024). 38263181 10.1038/s41597-023-02421-7 PMC10805868

[100] Wilke, M., Schmithorst, V. J. & Holland, S. K. Normative pediatric brain data for spatial normalization and segmentation differs from standard adult data. *Magn. Reson. Med.* **50**, 749–757 (2003). 14523961 10.1002/mrm.10606

[101] Franke, K., Luders, E., May, A., Wilke, M. & Gaser, C. Brain maturation: predicting individual BrainAGE in children and adolescents using structural MRI. *NeuroImage* **63**, 1305–1312 (2012). 22902922 10.1016/j.neuroimage.2012.08.001

[102] Wilke, M., Holland, S. K., Altaye, M. & Gaser, C. Template-O-Matic: a toolbox for creating customized pediatric templates. *NeuroImage* **41**, 903–913 (2008). 18424084 10.1016/j.neuroimage.2008.02.056

[103] La Rosa, F. et al. BrainAgeNeXt: advancing brain age modeling for individuals with multiple sclerosis. *Imaging Neurosci*. **3**, imag_a_00487 (2025). 10.1162/imag_a_00487 PMC12319810 40800843

