# Supplementary material for: Current challenges and future directions for brain age prediction in children and adolescents
Source: Nat Commun. 2025 Aug 20;16:7771. doi: 10.1038/s41467-025-63222-7 (PMC12368027; doi:10.1038/s41467-025-63222-7)
Supplement: Supplementary file 1 — Supplementary Infomation [file 41467_2025_63222_MOESM1_ESM.pdf]

Supplementary Table 1. Overview of selected brain-age prediction studies in children and adolescents.

Authors	Title	Age Range (Training)	Age Range (Testing)	Cohort (Training)	Cohort (Testing)	Model Performance (reported in MAE of years unless otherwise specified)	r/R^2	Features	Model Type Used	Outcome
Ball et al., 2017	Modelling neuroanatomical variation during childhood	3-21	3-21, 5-21	PING	ABIDE	1.54; 1.65 (ABIDE)	--	Brain tissue volume, cortic	Neighborhood preserving emb	--
Ball et al., 2021	Individual variation underlying brain age estimates in t	3-21	3-21	PING	PING	1.81, 1.75, 1.92	R^2 = 0.79, 0.81, 0.78	Cortical thickness, area	regularised linear regression (e	cognition
Beck et al., 2024	Dimensions of early life adversity are differentially ass	8.9-13	8.9-13	ABCD	ABCD	MAE: 0.79 (T1), 0.71 (DTI), 0.89 (	R^2: 0.34 (T1), 0.43 (DTI), 0.17	T1 (cortical surface area, th	XGBoost	trauma/adversity
Brouwer et al., 2021	The Speed of Development of Adolescent Brain Age D	9-23	9-23	BrainSCALE	BrainSCALE	1.24 (GMD model), others availab	R^2 = 0.77	(1) a voxel-based GMD fea	SVR (support vector regressio	genetics, sex differences
Brown et al., 2012	Neuroanatomical Assessment of Biological Maturity	3-20	3-20	PING	PING	1.71 (T1), 1.71 (DTI), 1.6 (T2)	R^2: 0.828 (T1), 0.806 (DTI), 0.	Cortical thickness and area	regularized multivariate nonlin	--
Cao et al., 2015	Development and validation of a brain maturation inde	4-18	6-17	NIH Pediatric MRI Data Re	NIH Pediatric MRI Data I	1st timepoint: 1.69, 2nd timepoint:	1st timepoint: R: 0.82, 2nd time	Cortical & subcortical volur	LASSO	--
Chung et al., 2018	Use of Machine Learning to Determine Deviance in Ne	3-21	12-21	PING	North American Prodrom	1.69, 1.41 (external, NAPLS2)	R^2: 0.84 (PING), 0.51 (NAPLS	Cortical volume, subcortica	Ridge regression	psychosis
Cohen et al., 2024	Relative Brain Age Is Associated with Socioeconomic	5-18	5-18	HBN	HBN	1.41 (may refer to original model c	R^2 = 0.71 (may refer to origina	Cortical mean curvature, w	JIVe, Ridge regression	SES, anxiety, depression
Cropley et al., 2021	Brain-Predicted Age Associates With Psychopathology	8-21	8-21	PNC	PNC	1.49, 1.61 (non-typically developir	r = 0.82 (TD), 0.72 (non-TD)	Cortical volume, area, thicl	SVR (support vector regressio	ADHD
Dehestani et al., 2023	Developmental brain changes during puberty and ass	9-13	9-13	ABCD, (HCP for suppleme	ABCD	7.5 months	r = 0.47	Cortical volume, area, thicl	SVR (support vector regressio	puberty, mental health
de Nooij et al., 2020	Longitudinal trajectories of brain age in young individu	15-28	15-30	Scottish Bipolar Family Stu	Scottish Bipolar Family S	2.21	r = 0.4	smoothed modular grey ma	RVR (relevance vector regres	mental health
Drobonin et al., 2022	The Developmental Brain Age Is Associated With Adv	9-19	9-19	ABIDE, ABIDE-II, CMI, CC	FORBOW	1.53; 1.55/1.98 bias-corrected (val	--	Cortical volume, surface ar	XGBoost	depression, functional impairment, environmental adversity
Erus et al., 2015	Imaging Patterns of Brain Development and their Rela	8-22	8-22	PNC	PNC	1.22	r = 0.89	Gray (GM) and white (WM)	SVR (support vector regressio	cognition
Franke et al., 2012	Brain maturation: Predicting individual BrainAGE in ch	5-18	5-18	Pediatric MRI Data Reposi	Pediatric MRI Data Repo	1.1	r = 0.93	GM, WM, and CSF	RVR (relevance vector regres	preterm birth
Gotlib et al., 2022	Effects of the COVID-19 Pandemic on Mental Health a	18-75	13-17	ENIGMA	In-study sample	--	--	Cortical thickness and surfa	Ridge regression	covid-related cohort differences
Gottschewsky et al., 2024	Menarche, pubertal timing and the brain: female-speci	15.25 ± 3.6	11.91 ± 0.6	PNC	ABCD	MAE: 1.1, RMSE: 1.3	--	Cortical volume, area, thicl	XGBoost	menarche status, puberty
Hedderich et al., 2021	Increased Brain Age Gap Estimate (BrainAGE) in You	11-70	25-28	fCONN, NIH, IXI, C-MIND	Bavarian Longitudinal St	MAE and RMSE not reported (refe	--	GM, WM, and CSF	RVR (relevance vector regres	cognition, perinatal variables, prematurity
Holm et al., 2023	Linking brain maturation and puberty during early adol	5-93	9-13	21 publically available data	ABCD	.7, 1.4 (baseline, follow-up)	--	T1-weighted data	CNN (convolutional neural net	puberty
Karolis et al., 2017	Volumetric grey matter alterations in adolescents and	9-65	Adolescent	ABIDE, COBRE, OASIS, I	In-study sample	--	r = 0.81, 0.42 and 0.81	GMVs, LIs, GMVs & LIs. G	LASSO	premature birth
Keding et al., 2021	Differential Patterns of Delayed Emotion Circuit Matur	8-18	8-18	In-study sample	In-study sample	1.66 (whole-brain), 1.602 (emotior	r = 0.677 (whole-brain), 0.663 ((	Cortical and subcortical vol	stacked generalization	internalizing behavior
Keding et al., 2024	Diverging Effects of Violence Exposure and Psychiatri	8-24	8-24	PNC	PNC	MAE provided separately for each	R^2 provided separately for eac	rsfMRI	CNN (convolutional neural net	violence exposure, internalizing, externalizing
Kelly et al., 2022	Investigating brain structural maturation in children an	3-21	7, 13	PING	Victorian Infant Brain Stu	1.72 (PING), 1.92; 1.53 (ViBES, 7	r = 0.9 (PING)	Cortical volume, area, thicl	Gaussian process regression	preterm birth
Khundrakpam et al., 2015	Prediction of brain maturity based on cortical thickness	12.9 ± 3.8	12.9 ± 3.8	Pediatric MRI Data Reposi	Pediatric MRI Data Repo	1.95, 1.79, 1.74, 1.71, 1.68 (differe	r = 0.78, 0.81, 0.82, 0.83, 0.84 (	Cortical thickness	linear regression (elastic net)	cognition
Kurth et al., 2022	Preliminary evidence for a lower brain age in children	5-22	6-18	Pediatric MRI Data Reposi	In-study sample	--	--	Processed gray matter ima	RVR (relevance vector regres	ADHD
Lewis et al., 2018	T1 white/gray contrast as a predictor of chronological a	3-22	3-22	NIHPD, PING	NIHPD, PING	MAE: 1.52 (contract + thickness).	r = 0.92 (contrast + thickness)	T1 white/gray contrast, cor	linear regression (elastic net)	cognition
Luna et al., 2021	Maturity of gray matter structures and white matter co	5-21	5-21	HBN	HBN (Healthy Brain Netw	1.18, 0.26 (age-harmonized)	--	Thickness, volume, area, n	ensemble, SEML was best	mental health, cognition
Lund et al., 2022	Brain age prediction using fMRI network coupling in yo	8-22	8-22	PNC	HBN (Healthy Brain Netw	PNC: 2.43 MAE, 2.93 RMSE. HBN	r =0.6 (PNC), 0.54 (HBN)	rsfMRI	SLM (shrinkage estimation of	mental health
MacSweeney et al., 2024	Multimodal brain age indicators of internalising probler	9-13	9-13	ABCD	ABCD	Same as Beck et al., 2024	Same as Beck et al., 2024	Same as Beck et al., 2024	XGBoost	internalizing symptoms
Mareckova et al., 2023	Longitudinal study of epigenetic aging and its relations	6-89	Adolescen	ABIDE, CoRR, DLBS and	VULDE	--	--	Cortical thickness	RVR, gaussian process	epigenetic age
Ng et al., 2024	Frontoparietal and salience network synchronizations	7-30	7-30	In-study sample	In-study sample	MAE: 2.94, RMSE: 4.15	R^2 = .519	Task-based fMRI network c	Gradient Boosting	math performance
Nielsen et al., 2019	Evaluating the Prediction of Brain Maturity From Func	7-31	7-31	In-study sample	In-study sample	--	R^2: 0.57	rsfMRI	SVM	motion
Niu et al., 2022	Multidimensional brain-age prediction reveals altered I	14.41 ± 3.8	Healthy co	PNC	PNC	1.751 to 3.16 (5 sex-specific mode	R^2: 0.131 to 0.798 (5 sex-spec	Grey matter volume, FA	Ridge regression	Specific phobia, social phobia, depression, PTSD, ODD, ADHD
Rakesh et al., 2021	Neighborhood disadvantage and longitudinal brain-pre	8-21	12, 16, 19	PNC	OADS (Orygen Adolesce	Training (PNC): 1.43, Testing (OA	Training (PNC): r = 0.79, Testin	Cortical volume, area, thicl	SVR (support vector regressio	neighborhood disadvantage, parenting, effortful control
Ray et al., 2024	Adolescent brain maturation associated with environm	8-22	9-10	PNC, ABCD	ABCD	0.49	-	Grey matter density (ICA c	SVR (support vector regressio	cognition, and a set of environmental variables: air pollution, population density, area crime, neighborhood safety, school safety
Rudolph et al., 2017	At risk of being risky: The relationship between “brain a	10-25	10-25	--	--	Training RMSE: 1.503, Test RMSE	Train R^2: 0.81, test r^2: 0.42	pseudo-rsfMRI, cortical thic	PLSR (partial least squares re	risk perception and preference
Sanders et al., 2024 (preprint)	Associations between methylation age and brain age i	18-75	18-24	ALSPAC (Avon Longitudin	ALSPAC (Avon Longitud	4.3	r = 0.27	Cortical thickness and surfa	Ridge regression	methylation, physical, cognitive and mental health
Soehner et al., 2023	Naturalistic Sleep Patterns are Linked to Global Struct	18-92	9-25	brainageR; 7 cohorts: AIBL	NAPS (Neuroimaging an	3.933	r = 0.97	PCA from GM, WM, and C	Gaussian process regression	sleep
Stratton et al., 2024	Transient patterns of advanced brain ageing in female	12-23	12-23	NIH MRI Study of Normal	In-study sample	1.33 (grey matter); 0.97 (white ma	r = 0.81 (grey matter); 0.91 (wh	Grey matter, white matter	RVR (relevance vector regres	Anorexia nervosa (multiple clinical measures)
Truelove-Hill et al., 2020	A Multidimensional Neural Maturation Index Reveals F	8-21	8-21, 16-22	PNC	PING, multisite schizoph	sNMI: 1.67 (PNC), 1.59 (PING); fN	r (sNMI) = 0.822 (PNC), 0.872 (	Grey matter volume, white	SVR (support vector regressio	schizophrenia
Tunç et al., 2019	Deviation from normative brain development is associ	6-25	6-25	In-study sample	In-study sample	--	r = 0.88 (training sample), r=0.8	Cortical volume, area, thicl	SVR (support vector regressio	autism symptoms
Ullman et al., 2017	Timing of White Matter Development Determines Cog	6-20	6.8 ± 0.3	In-study sample	In-study sample	--	r = 0.81	FA	SVR (support vector regressio	Cognition
Wang et al., 2021	Predicting brain age during typical and atypical develo	8-23	5-30	PNC	Functional Connectome	1.1 to 1.47 (2 sex-specific models	r = 0.74 to 0.9 (2 sex-specific m	Grey matter volume, white	Partial least square regression	ASD
Whitmore et al., 2023	BrainAGE as a measure of maturation during early ad	9-19; 9-11;	9-14	Same as Drobonin et al., 2	ABCD	Existing model: 2.32 (uncorrected,	--	Cortical volume and area, s	XGBoost	puberty, cognition
Wierenga et al., 2019	Sex Effects on Development of Brain Structure and E	8-26	8-26	BrainTime	BrainTime	2.015	0.79 (adjusted R^2)	Cortical surface area & thic	Random forest	cognition, sex differences
Zhao et al., 2019	Brain age prediction: Cortical and subcortical shape co	5-18	6-18	HBN	NKI-RS	1.41	R^2 = 0.71	Covariance: cortical mean	Ridge regression	IQ, sex
Zhao et al., 2023	Differential effects of generalized anxiety and separati	5-18	Average ag	HBN (CBIC site)	HBN (RUBIC site)	1.97 (discovery), 1.81 (validation)	r = 0.678 (discovery), 0.734 (va	Cortical thickness, surface	SVR (support vector regressio	anxiety

[illegible]
Families Overcoming Risks and Building Opportunities for Well-being, HBN: Healthy Brain Network, IXI: Information eXtraction from Images,NKI-RS: Nathan Kline Institute - Rockland Sample, OASIS: Open Access Series of Imaging Studies, PING: Pediatric Imaging, Neurocognition, and Genetics, PNC: Philadelphia Neurodevelopmental Cohort, VULDE:
